# Revealing text in a complexly rolled silver scroll from Jerash with computed tomography and advanced imaging software

**DOI:** 10.1038/srep17765

**Published:** 2015-12-09

**Authors:** Gry Hoffmann Barfod, John Møller Larsen, Achim Lichtenberger, Rubina Raja

**Affiliations:** 1 Institute for Geoscience, Aarhus University, Høegh-Guldbergs Gade 2, 8000 Aarhus C, Denmark; 2 Institute for Culture and Society, Aarhus University, Jens Chr. Skous vej 3, 8000 Aarhus C, Denmark; 3 Institute für Archäologische Wissenschaften, Ruhr-Universität Bochum, Am Bergbaumuseum 3144791 Bochum, Germany

## Abstract

Throughout Antiquity magical amulets written on papyri, lead and silver were used for apotropaic reasons. While papyri often can be unrolled and deciphered, metal scrolls, usually very thin and tightly rolled up, cannot easily be unrolled without damaging the metal. This leaves us with unreadable results due to the damage done or with the decision not to unroll the scroll. The texts vary greatly and tell us about the cultural environment and local as well as individual practices at a variety of locations across the Mediterranean. Here we present the methodology and the results of the digital unfolding of a silver sheet from Jerash in Jordan from the mid-8^th^ century CE. The scroll was inscribed with 17 lines in presumed pseudo-Arabic as well as some magical signs. The successful unfolding shows that it is possible to digitally unfold complexly folded scrolls, but that it requires a combination of the know-how of the software and linguistic knowledge.

In Antiquity thin metal sheets (*lamellae*) of various compositions (lead, silver, gold or an alloy) were used for writing magical or apotropaic texts on^1^,^2^,^3^. This practice was longstanding in the Classical world and was an established way in which uncertainties of daily life were dealt with, including cases of illness, unrequited love and as protection against the evil eye, just to mention a few. Such texts therefore let individuals speak directly to us through a medium and they give vivid insight into ancient mentalities and emotions, an insight which we hardly get from other literary or official ancient texts such as historiography, poetry or inscriptions.

In most cases the script is incised into the surface of the metal tablets and only rarely on wooden tablets^4^. Usually the metal tablets were folded or tightly rolled up after the texts had been written on them and they were then placed in an outer case^5^. The tablets were not intended for being unrolled and read again, but fulfilled their (magical) function once encased in the container. Such objects could for example be deposited in houses or carried on the body for protection. They were personal belongings made for a specific occasion. Due to the fragility of the thin material, it is often difficult or impossible to unroll and decipher the sheets without destroying them and numerous specimens are kept in museum storages around the world. Computed tomography has in the case of one Late Antique Mandaean lead scroll from the 5^th^ century CE been used to digitally unroll a metal scroll^6^. The digital unrolling in this case was undertaken by Henry Weber and Katrin Lück with advanced imaging software (VGStudio MAX 2.2) and focused on defining polylines along which the scroll could be digitally unrolled. The unrolling of the entire scroll was possible with this program because the scroll was regularly rolled up and not deformed.

This technique combining computed tomography and general purpose industrial imaging software for voxel analysis was applied to a silver sheet recently found during excavations in Jerash, Jordan, which is presented here for the first time. Similar techniques have recently successfully been applied to ancient carbonized papyri from Herculaneum in the bay of Naples^7^,^8^. However, in these cases the different material of the scrolls as well as the use of a different writing technique (ink and not incision) results in a different problem and asks for other solutions and can therefore not be compared directly to the current case.

In this article we present a methodology for digitally unfolding irregularly folded and heavily deformed inscribed metal sheets, which may be applied to numerous finds yet to be studied. This methodology promises to optimize the access to knowledge accumulated in museum storages and excavation depots and makes it possible to unveil hidden ancient texts, which until now remain inaccessible due to complex folding or deformation. The methodology furthermore promises to unleash further cultural understanding, telling us about the continuity and change in the use of magical texts as protective measures, which was a phenomenon spanning from classical Greek Antiquity to the early Islamic period and to modern times across a large geographical area interconnected through historical developments, including conquests, changes in rule and trade relations. The methodology might furthermore be applied to other groups of complexly deformed objects such as metal vessels with decoration.

Jerash is an ancient urban site in Northwest Jordan with considerable Roman, Byzantine and Early Islamic remains. The city prospered in these periods. An earthquake in the year 749 CE brought an end to civic life and most parts of the city remained desolate until resettlement in the Circassian period in the 19^th^ century^9^. During excavations conducted by the Danish-German Jerash Northwest Quarter Project under the direction Achim Lichtenberger (Ruhr-Universität Bochum, Germany) and Rubina Raja (Aarhus Universitet, Denmark), a private dwelling was excavated in 2014^10^. The house, which held rich finds, had been destroyed by the earthquake in 749 CE as attested by pottery dated according to established typologies, numismatic finds, as well as through radiocarbon dating of charcoal in sealed contexts in the house. The inventory of the house remained undisturbed after the house was destroyed. It was in this context that the silver sheet in a container was found and it is securely dated to before 749 CE.

The silver amulet was folded, rolled and then placed in a cylindrical lead container (Fig. 1). A slice showing the complex rolling of the sheet is seen in Fig. 2, while Fig. 3 shows the entire scroll and the position of the slice. The metal composition of container and amulet were determined by electron microprobe (EMP) and laser ablation inductively coupled plasma mass spectrometry (LA-ICPMS) by Sarah Roeske and Gry Hoffmann Barfod at the University of California at Davis. These showed that the amulet was silver, while the container was made of lead with traces of presumably inlaid decoration in tin and ‘bronze’. Surrounding the lead was highly corroded material of mainly iron oxide, which we interpret to be secondary material. On the lead container there are also traces of textiles and threads. The positioning of the traces point to a contemporary wrapping of the iron container in textile, which thereafter was rolled up with thread. The traces of the threads are located approx. on the middle of the lead container. Conservator Helmut Franke, Potsdam, Germany conserved the object as far as possible and could despite heavy corrosion of the case remove the scroll from the case (Fig. 4). It was clear when the scroll was removed from the container that it was inscribed, since several lines of incised letters were visible on the reverse (which is the show side after the sheet was folded) of the folded metal sheet. However, it was not possible to unroll the scroll mechanically, since the metal was too thin and fragile and the danger was that the object would suffer irreversible damage. Therefore it was submitted to a high resolution CT scan (Phoenix GE v|tome|x M equipped with a 300 kV microfocus X-ray tube and a 2k imaging detector). The resulting data set (the volume) was then analyzed in the computer program VGStudio MAX by John Møller Larsen (Aarhus University, Denmark).

This analysis was made possible by the development of new software: In 2012 Volume Graphics added a function to their program VGStudio MAX, which made the virtual unrolling of the Mandaean lead scroll possible. This function allowed users of the program to freely define a set of polylines serving as guiding lines for such an unfolding. It was successfully applied to the Mandaean lead scroll. This scroll was, compared to the complexly folded and rolled scroll from Jerash, rolled regularly and had not been squeezed or deformed in any way.

The initial outset of the current project was to apply the same technique to the scroll from Jerash, but the scroll proved to be too complexly folded and rolled for this to be a viable option. Therefore the methodology had to be adapted to fit the case and it was decided to define each line as a ‘region of interest’ (ROI), i.e. as a separate segment. The computer program allows the selection of volume data (the volume pixels or *voxels*) by different means including a 3D draw tool. Much of the text could easily be selected, but in many cases, where the metal sheets touched each other, the segmentation was a considerably more time-consuming process. The fine ‘scratches’ which can be seen in the rightmost part of line 1 (Fig. 5) and in parts of line 10 (Fig. 6) are due to the draw tool.

When the volume had been segmented, it was possible to view each line of text in isolation and to view both the front (obverse) and the back side of the scroll (reverse). In most cases it turned out that the reverse, where the incised text appears in high relief, is the more legible one. For the deciphering the images of the writing seen on the obverse had to be mirrored.

The technique used for the unrolling of the Mandaean lead scroll could only be used to a limited extent for the scroll from Jerash. As an example, Fig. 7 shows line 9, where the letters to the right are only partly visible since the top of the letters are hidden around the bent in the sheet. In this case 30 polylines with a length of approx. 6 mm, each consisting of 30 points, were applied, also shown in Fig. 7. The result of the complete unrolling is seen in Figs 5 and 6. While this technique does not provide the clarity of the segmented images, it can certainly help in the production of the line drawings that accompany the philological edition of the text, and here it should be emphasized that the user is not limited to the unfolded version but can move the segment three-dimensionally in real time. In other words, manipulating the segment on the screen the user can establish what features constitute writing and which not, while the unrolled version can help in establishing the proportion of the letters.

## Results

### Sample characteristics

The case containing the silver scroll was found in a house in an earthquake sealed context dating to the mid-8^th^ century CE. The container was 1 mm thick, 50 mm long and had a diameter of 17 mm. There are no smithing lines visible on the container although these may be obscured by heavy corrosion and mineralization on the exposed surfaces. It had lost substance due to this corrosion and had several sediments, crustations and soil sticking to it. Furthermore the container had been deformed and had a crack running along its long side as well as several small fractures and places where parts of the metal had broken off. Backscattered electron (BSE) image for a 1 cm long fragment of the container revealed a ‘pristine’ metal core surrounded by dark material (Fig. 8a). Detailed coloured elemental maps for the area marked by a white box in Fig. 8a showed the pristine metal to be lead framed by tin (two upper coloured frames (Pb and Sn) in Fig. 8). The only other elements detected within the lead core were tiny specs of bronze-like material (Sn:Cu 3:2, Cu map in Fig. 8a and table 1). Elemental maps of Fe (Fig. 8b) and O showed the lead core to be ‘encased’ by iron oxide. Based on these observations we infer that the original container was made of lead with tin and tin-copper alloy decorations but that the iron oxide likely is a secondary feature deposited later from perculating groundwater.

**Figure 5:**
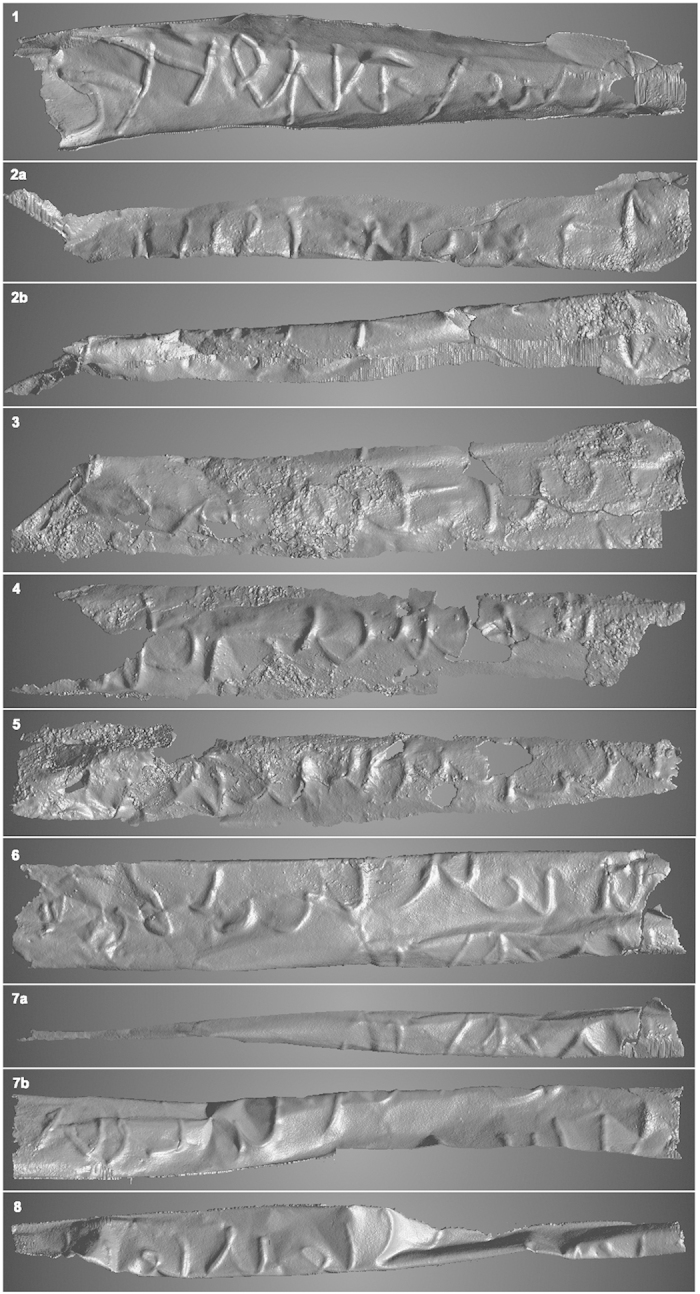
Lines 1–8 from the silver scroll; all back sides, mirrored. For lines 2 and 7 the upper and lower parts are shown as separate images (a,b).

The rolled silver scroll was approx. 0.15 mm thin and 42 mm long with a diameter of 8 mm. The metal sheet had a length of 9.5 cm. The letters on the scroll were originally incised with a fine stylus with a rounded tip. The letter size can be exemplified by the initial N-shaped sign in line 6 (fig. 5) which is approx. 3 mm × 5 mm. The scroll was corroded, mineralized on the surface and had lost smaller fragments off the edges.

The scroll could only be recovered from the Pb container after careful cleaning and restoration. As was the case for the container, the BSE images for a cross section of a scroll fragment display least corrosion of the inner part of the scroll (Fig. 8b). Quantitative analysis showed the core to be nearly pure silver with a minor gold component around 0.2 wt% (Fig. 8. Silver is a very noble metal and is often found in its native state. Thus it does not corrode easily, but after long exposure AgCl is the most common corrosion product^11^. The more altered parts of the scroll (darker areas in Fig. 8) had a higher proportion of silver and chlorine relative to the core suggesting corrosion has taken place from the edges. Besides silver, chlorine and gold the only other elements observed in the scroll were minor amounts of copper (Cu), aluminum (Al), lead (Pb), bismuth (Bi) and Rare Earth Element (represented by samarium (Sm) in Table 2).

## Reading

The sheet consists of 17 lines of text, most of which resembles Arabic. In these parts the script is ligatured, unvocalized and without diacritical points. It is, however, difficult to identify meaningful words, and there certainly are many cases that can hardly be reconciled with Arabic, both in regard to letter shapes and ligaturing. The text contains a striking number of letter forms based on a vertical stroke, and considering the length of the text, it is notable that we find no clear instances of the otherwise commonly used letters ج/ح/خ, ص/ض, ط/ظ, ع/غ, ك, م, ه. In view of these difficulties, the question arises whether the script in fact is pseudo-Arabic, i.e. it mimics parts of the graphical repertoire of Arabic script but does not convey a meaningful text and on occasion employs shapes that are hard to reconcile with Arabic letters. Pseudo-script is a well-known phenomenon in magical texts; for instance, we have numerous examples in the Aramaic incantation bowls^12^,^13^. In regard to pseudo-Arabic, research has mostly dealt with specimens from Europe and Byzantium^14^, but the phenomenon is widely found in the Muslim world, too^15^.

**Figure 6:**
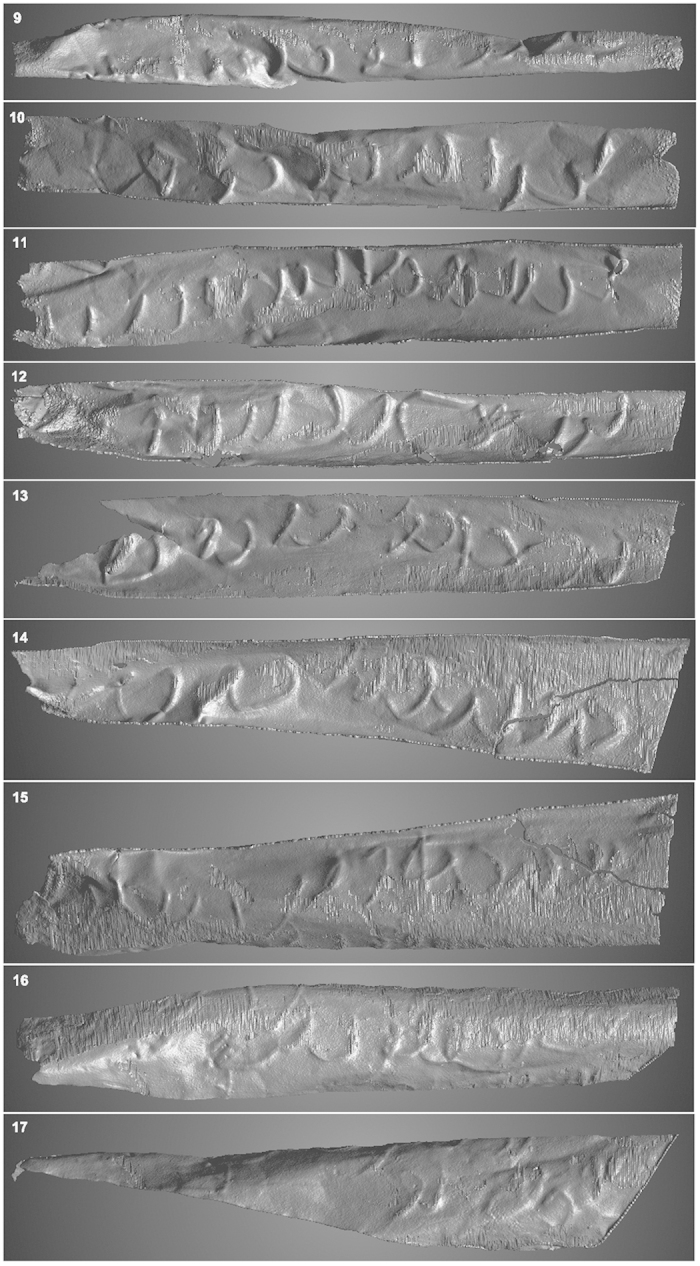
Lines 9–17 from the silver scroll; all back sides, mirrored.

The text also contains signs which clearly are not Arabic or imitate Arabic letters. Most of these occur in line 1 (Fig. 5), i.e. the seven signs to the left. A few further signs are found in some of the following lines (Fig. 5, lines 2, 5, 6 and Fig. 6, lines 10 and 17). Collectively, it has not been possible to assign these signs to a known alphabet. Since we presume that the artefact is an amulet, and since the main text seems to be in pseudo-script (characteristic of many magical texts), it is reasonable to view the signs as ‘magical’ (charaktêres). The use of magical signs was widespread in Antiquity and they are found in texts written on a multitude of mediums. In Greek, for instance, we have many examples from papyri, from metal amulets^16^, and from engraved gems. We have examples on Aramaic amulet texts incised on metal sheets^17^,^18^, and on occasion, magical signs are also found on Aramaic incantation bowls^19^,^20^. Frequently, such signs are of the ‘ring-letter’ type (*Brillenbuchstaben*), signs characterized by the addition of small rings or loops at the end of lines, but they can have numerous different forms, and the signs here belong to the simpler, unadorned forms^21^. The use of magical signs in Antiquity greatly influenced the Jewish, Christian and Muslim magical traditions^22^,^23^.

The interpretation of early Arabic texts often involves some special problems. As in modern texts, short vowels are rarely indicated, while long vowels usually are expressed by means of letters which also serve as consonants. In the developed Arabic script, certain letters are distinguished only by the use of diacritical points, for instance the letters ب (*bā ,*), ت (*tā ,*) and ث (*thā ,*). With no diacritics, as in this case, several interpretations are often possible. In addition, some Arabic letters have a considerable similarity, increasing the number of readings even further. The following tentative readings show the difficulty of arriving at a meaningful interpretation.

Line 6 (Fig. 5) begins with an N-shaped letter, possibly a magic sign as in line 1 (Fig. 5). Following this we may read. Since the first letter has no diacritics, there are five possible interpretations 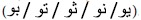. It is noteworthy that this combination is found in lines 7–11 and 17 as well. The top of the following letters go slightly around the bend in the sheet. From a different angle (Fig. 9) we can see the complete letters, although the proportions are now somewhat distorted. It would be possible to read this either as an N-shaped sign with a long and curved left leg (and thus not as Arabic) or as 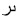, where the first letter would have the same five possibilities as above, and the second letter could be read as ر or ز. The next letters could be read 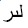, but without diacritics the second letter has the same five possibilities as above, while the third letter again can be either ر or ز. This goes for the next letter too. We then have an ا, but the following is difficult. It could possibly be read as a *lām*-*alif* ligature, but then we cannot explain why the following ر or ز has a ligature. Finally, the sign to the left is difficult to interpret as Arabic, unless it is a poorly written *lām*-*alif* ligature.

In line 10 (Fig. 6) the initial letters may be read as 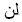. If so, the following letter (ر or ز) merely touches the ن and does not form a proper ligature (this is of course only true under the assumption that the writer followed the normal rules of orthography). Following this we have an ا. The next two letters are difficult. The first letter could be a ل in its isolated form; it does not form a ligature to the second letter which could be ر or ز if it were not placed *above* the base line. The next could again be read as a N or as 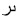. Following this we have the recurrent 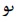. Finally, to the left we probably have a magic sign.

## Discussion

The application of the combination of a high resolution CT scan and general purpose industrial imaging software for voxel analysis (VGStudio MAX 3 Beta) on a heavily deformed and folded inscribed metal sheet allowed the digital unfolding. Whereas earlier applications of this technique only were used for reconstructing regularly rolled scrolls, it has now been shown that it is possible to also reconstruct objects, which are complexly deformed or folded. The resolution offered by the scan provided a more than adequate quality to render clear images, and the use of advanced 3D imaging software has many other advantages. For instance, the artefact can be turned and twisted in real time by the user, who also has complete control over the illumination. One or more virtual light sources can be set up and moved around to manipulate light and shadow, bringing out otherwise hidden details. It, however, has to be emphasized that this way of reconstructing the ancient texts is very time consuming.

The combination of the find and the methods gave a unique opportunity to test the limits of the implemented methodology. Overall the results were satisfactory and promising for further research. It showed that this technique can be applied to much more complex situations than thought. The technique of isolating lines through segmentation has generally provided good results. The main difficulty is when two sheets are in physical contact, which makes the segmentation intricate work, which is extremely time consuming and can result in producing the fine ‘scratches’ seen in some of the unfolded lines. In spite of the highly detailed 3D image data created by the CT scan, the volume has a size of approx. 4GB which can be analyzed on average PC and laptop systems. The segmentation process generally went smoothly, while unrolling by means of polylines put a greater strain on the ordinary computer systems which were used once a large number of polylines had been added. Further studies and more experience with the imaging software may well provide improved results in the future. In addition to segmentation, this study has showed that virtual unrolling can be a useful supplementary technique in cases where the metal sheet is crumbled or a line of text happens to coincide with a bend in the scroll.

The impact of the discovery that complexly folded scrolls can be unfolded successfully with the technique applied here has crucial implications for all future studies of such objects, which have remained unfolded. Firstly it calls for a holistic approach to the objects combining software knowledge with philological expertise. Secondly it opens completely new perspectives on the research of such objects, which until now largely have remained closed land for scholars. In the case of the Jerash scroll it has been possible to visualize the ancient script on the silver sheet. Although the incised letters did not form a readable text, they provide us with important information on the cultural milieu in which this amulet was produced. Earlier finds have documented Arabic texts in the early Arab period in Jerash and the use of Arabic (at least for some purposes) by both Christians and Muslims^24^,^25^. The use of what is presumed to be Arabic pseudo-script shows that it was deemed suitable for magical purposes as well. Since it was not possible to read the ‘text’ we cannot identify the religious affiliation of the amulet’s owner. Such pseudo-scripts and letter-like magical signs are furthermore important evidence for literacy or non-literacy in the ancient world and the value and power written texts had for people. Therefore even if in the case of the Jerash scroll we cannot reconstruct a text from the (pseudo-)script, it is an important contribution to the reconstruction of mentalities and daily life in the ancient world. The application of the new technique on other hitherto unfolded metal sheets (such as curse-tablets) will provide us with further glimpses into the mentalities of people in the ancient and medieval worlds.

## Methods

### Scroll and container fragments were mounted under vacuum in epoxy, sectioned, and polished.

**Figure 8:**
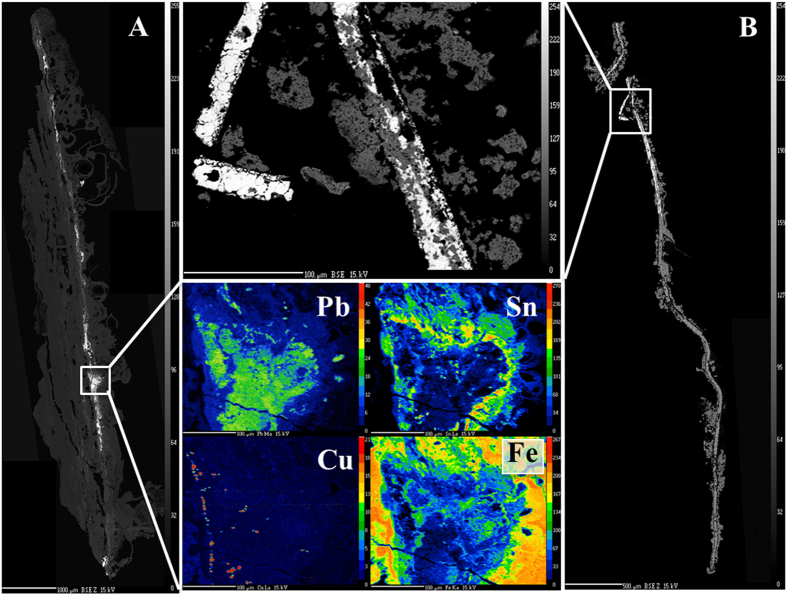
(**a**) Backscattered electron (BSE) image of container fragment (about 1 cm in length), showing ‘pristine’ lead surrounded by replaced (?) iron oxide. The four colored panels show detailed elemental maps of lead (Pb), tin (Sn), copper (Cu) and iron (Fe) for the cross section area marked by box on BSE image. (**b**). BSE image of scroll fragment. White areas are silver, and darker areas silver chloride. Detailed BSE image of silver (upper, middle) corresponds to the box on the overview BSE image. Quantitative WDS spot analyses were done in this area.

**Figure 9:**
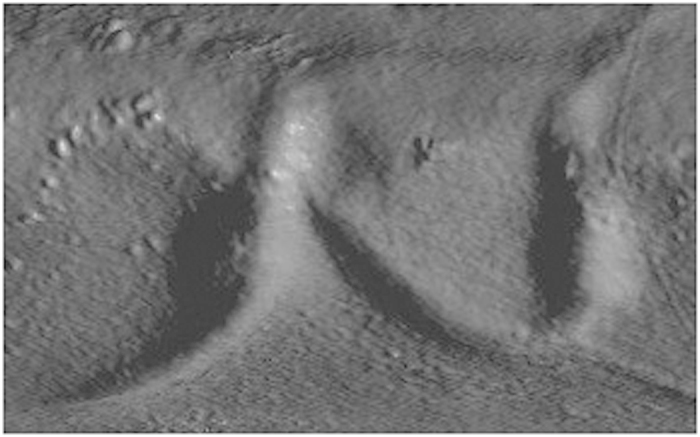
Detail from line 6, back side (line 6 is also shown in Fig. 5).

**Scanning electron microprobe (EMP)** at the Electron Microprobe Laboratory of the Department of Earth and Planetary Science, University of California at Davis was used to create elemental maps and BSI images as well as for quantitative analyses. The CAMECA SX-100 electron microprobe (EMP) is equipped with five wavelength dispersive spectrometers (WDS) and high-resolution, back-scattered electron (BSE) imaging. The analytical conditions were an acceleration voltage of 15 kV, a beam current of 10 nA and spot sizes of 1 μm. Hematite, copper and tin metals as well as PbS were analyzed concurrently as controls and measure of precision and accuracy. The analytical precision is about 3% for major elements and increases for low concentrations to 5% or above, with a lower limit of detection for the latter at 0.01 wt. % (Fig. 8).

**LA-ICPMS trace element analysis** were done at the Center for Plasma Mass Spectrometry, University of California at Davis analyses using a Agilent Technologies 7500a quadrupole ICP-MS coupled to a New Wave Research UP-213-nm laser with He as the carrier gas. Laser parameters were set at 60% energy, 10 Hz pulse frequency, and 10–20 μm spot size. USGS synthetic glass standard GSE-G1 was used as calibration standard and GSD-1G was run repeatedly (n = 6) as an unknown showing reproducibility within 5% or less of certified values^26^. Pb and Ag were used as internal standards for the container and scroll analysis, respectively.

### Computer tomography

The computed tomography was conducted by the company Vohtec in Garching at Munich in February 2015. Vohtec used a scanner normally employed for industrial purposes and for the inspection of building parts, the Phoenix GE v|tome|x M equipped with a 300 kV microfocus X-ray tube and a 2k imaging detector. The scan was done at an X-ray voltage of 270 kV and a current of 87 μA. The imaging set up produced a magnification of approx. 8,5x with a voxel size of approx. 23 μm. The 3D volume was then created on the basis of 1,600 2D X-ray images recorded during the scan as the object was rotated 360° around its main axis.

### Software processing, VGStudio MAX 3 Beta

The segmentation of the entire volume and the unrolling of smaller parts were done using a beta version of VGStudio MAX 3 developed by Volume Graphics in Heidelberg.

## Additional Information

**How to cite this article**: Hoffmann Barfod, G. *et al.* Revealing text in a complexly rolled silver scroll from Jerash with computed tomography and advanced imaging software. *Sci. Rep.* **5**, 17765; doi: 10.1038/srep17765 (2015).

## Figures and Tables

**Figure 1:**
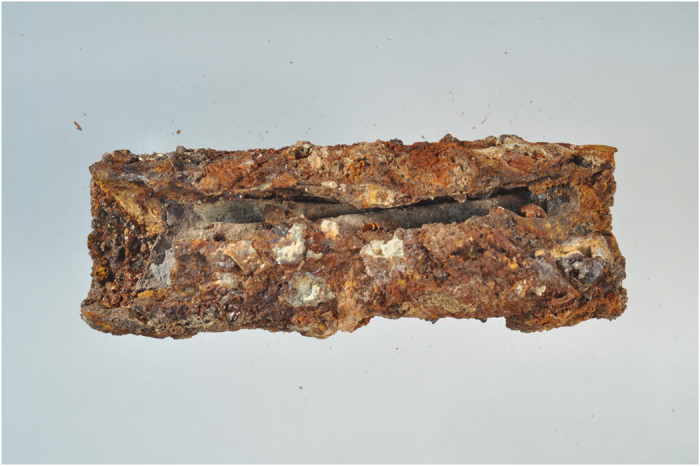
Silver scroll enclosed in lead container before conservation. The silver scroll is visible through the break in the lead case. The scroll has a light greyish color, partly due to corrosion. The lead container is surrounded by highly corroded material of mainly iron oxide and other inorganic materials, such as small stones.

**Figure 2:**
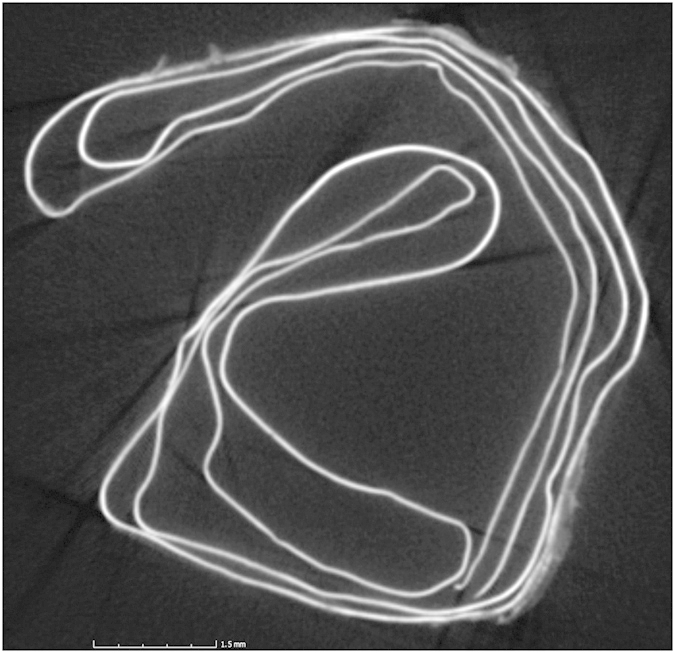
Slice showing the folding of the silver sheet.

**Figure 3:**
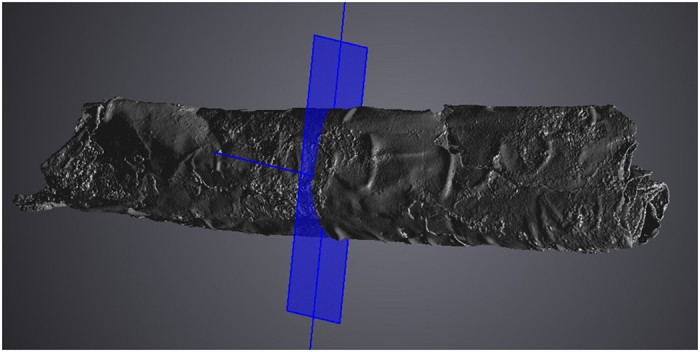
The scroll rendered in VGStudio MAX 3 Beta with indication of the slice shown in Fig. 2.

**Figure 4:**
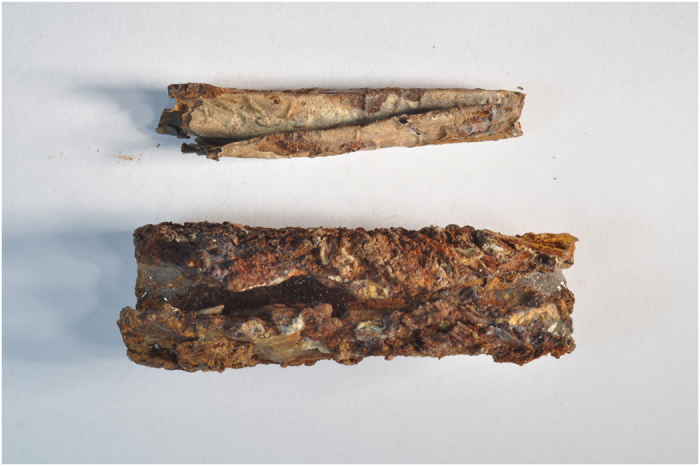
Silver scroll (above) and lead case (below) after separation by conservator. On the silver scroll several lines of writing are visible.

**Figure 7:**
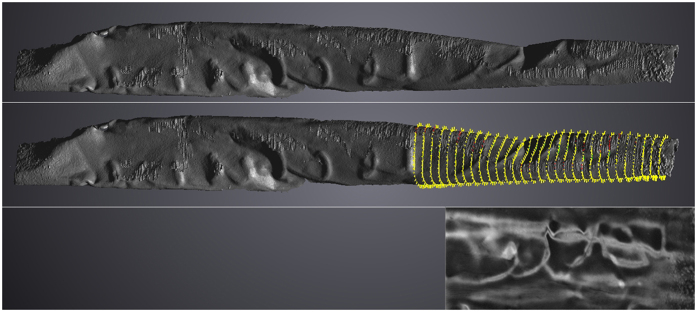
Line 9, back side, shown before and after the addition of polylines. Below: The right part of line 9 digitally unrolled.

**Table 1:** Bulk composition (wt%) by scanning electron microprobe (EMP) for high Pb, Sn and Cu areas within the container core shown in Fig. 8a.

Container	Mean	Std dev	Container	Mean	Std dev	Container	Mean	Std dev
Core−Pb	n = 4	n = 4	Core−Sn	n = 3	n = 3	Core−Cu	n = 3	n = 3
***wt*****%**			***wt*****%**			***wt*****%**		
**Pb**	72.01	0.57	**Pb**	18.09	5.11	**Pb**	3.11	4.69
**Sn**	0.80	0.28	**Sn**	39.93	0.19	**Sn**	58.54	3.28
**Cu**	BDL		**Cu**	0.19	0.04	**Cu**	38.39	4.04
**Fe**	0.76	0.47	**Fe**	8.48	1.53	**Fe**	0.52	0.17
**Total**	72.50	0.82	**Total**	66.69	3.81	**Total**	100.55	1.50

All other elements are below detection limit. n–number of analyses; std dev–standard deviation; wt%-weight percent.

**Table 2:** Major element composition (wt%) by EMP and trace element composition (ppm) by LA-ICPMS for scroll.

	Scroll	std dev
	Mean	
**EMP**	n = 8	n = 8
***wt*****%**		
**Ag**	100	0.50
**Cl**	0.18	0.10
**Au**	0.19	0.02
**Total**	100.5	0.48
	**Mean**	**std dev**
**LA ICP**	**n = 4**	**n = 4**
***ppm***		
**Al**	715	11.0
**Cu**	748	110
**Sm**	54.1	3.81
**Au**	1921	111
**Pb**	163	3.12
**Bi**	99.4	12.3

All other elements are below detection limit. Detection limits for chlorine (Cl) and gold (Au) for EMP analysis are 0.02 and 0.1 wt%, respectively. ppm - parts per million. Other abbreviations as in Table 1.
